# Using GIS Mapping of the Extent of Nearshore Rocky Reefs to Estimate the Abundance and Reproductive Output of Important Fishery Species

**DOI:** 10.1371/journal.pone.0030290

**Published:** 2012-01-17

**Authors:** Jeremy T. Claisse, Daniel J. Pondella, Jonathan P. Williams, James Sadd

**Affiliations:** 1 Vantuna Research Group, Department of Biology, Occidental College, Los Angeles, California, United States of America; 2 Department of Geology, Occidental College, Los Angeles, California, United States of America; University of Bristol, United Kingdom

## Abstract

Kelp Bass (*Paralabrax clathratus*) and California Sheephead (*Semicossyphus pulcher*) are economically and ecologically valuable rocky reef fishes in southern California, making them likely indicator species for evaluating resource management actions. Multiple spatial datasets, aerial and satellite photography, underwater observations and expert judgment were used to produce a comprehensive map of nearshore natural rocky reef habitat for the Santa Monica Bay region (California, USA). It was then used to examine the relative contribution of individual reefs to a regional estimate of abundance and reproductive potential of the focal species. For the reefs surveyed for fishes (i.e. 18 out of the 22 in the region, comprising 82% the natural rocky reef habitat <30 m depth, with a total area of 1850 ha), total abundance and annual egg production of California Sheephead were 451 thousand fish (95% CI: 369 to 533 thousand) and 203 billion eggs (95% CI: 135 to 272 billion). For Kelp Bass, estimates were 805 thousand fish (95% CI: 669 to 941thousand) and 512 billion eggs (95% CI: 414 to 610 billion). Size structure and reef area were key factors in reef-specific contributions to the regional egg production. The size structures of both species illustrated impacts from fishing, and results demonstrate the potential that relatively small increases in the proportion of large females on larger reefs could have on regional egg production. For California Sheephead, a substantial proportion of the regional egg production estimate (>30%) was produced from a relatively small proportion of the regional reef area (c. 10%). Natural nearshore rocky reefs make up only 11% of the area in the newly designated MPAs in this region, but results provide some optimism that regional fisheries could benefit through an increase in overall reproductive output, if adequate increases in size structure of targeted species are realized.

## Introduction

**Figure 1:**
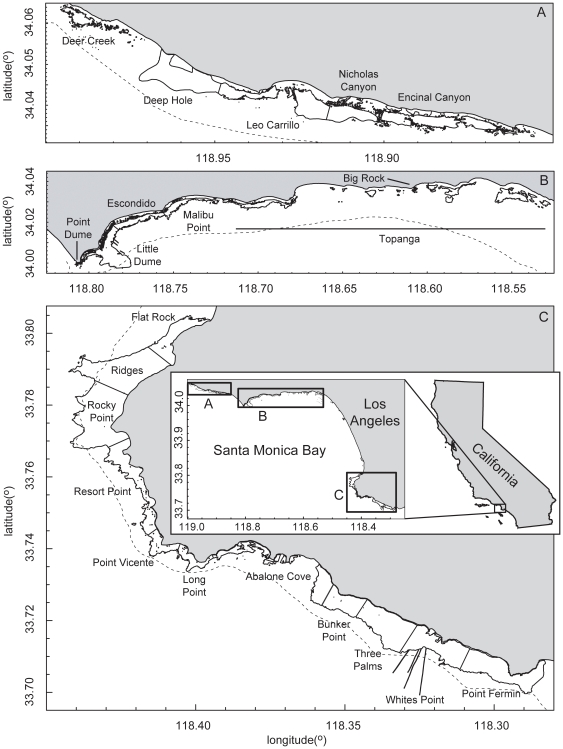
Extent of nearshore rocky reefs in Santa Monica Bay, California. Extent of nearshore rocky reefs in Santa Monica Bay, California for (A) western Malibu, (B) eastern Malibu and (C) Palos Verdes Peninsula. The 30 m depth contour appears as a dotted line. Note that while some artificial reefs (for example sewer outfalls at Whites Point) and natural rock reef habitat extending below 30m are included here, they were not included in the area estimates (total extent of rocky reef habitat <30 m depth) in Table 1.

In the nearshore marine environment of southern California, rocky reef habitats are a primary limited resource [1]. An estimated 15% to 25% of the mainland coast of California south of Point Conception is rock, separated by large stretches of sandy beach [2], [3]. However, these rocky reefs and the associated forests of giant kelp (*Macrocystis pyrifera*) support a higher diversity and abundance of fishes than most other marine habitats in the region [1]. They are critical habitat for many commercial and recreational fisheries species, including many that now have protected or endangered status [e.g., giant sea bass (*Stereolepis gigas*) [4], multiple abalone species (*Haliotis sorenseni*, *H. cracherodii*, *H. corrugata* and *H. fulgens*) [5]. At the heart of this region is Santa Monica Bay, which has two major rocky headlands, Malibu and the Palos Verdes Peninsula (Figure 1). The marine resources in this area support numerous economic interests, and with a growing population, the nearshore rocky reefs are impacted by a variety of local anthropogenic stressors (e.g., overfishing, turbidity, sedimentation, pollution) [6]–[10]. Commercial and recreational fisheries in southern California have greatly affected the abundances, size distributions, and/or spatial distributions of numerous fish and invertebrate species [10]–[15]. However, a management action prohibiting the nearshore use of gill nets in this region resulted in a return and increase in density of large predatory fishes, demonstrating the ability of resource managers to positively influence this system [16]. Other major spatial management actions (e.g., marine protected areas, habitat restoration) are currently being implemented in the region [17]. Despite the importance of rocky reef habitat, their extent and location had yet to be thoroughly mapped in this area - a critical caveat when considering their contribution to the subtidal nearshore environment.

**Table 1:** Reef areas and summary of depth zone specific fish sampling.

			Transect Count					Proportion of Reef			
	Reef	Area (ha)	Inner	Middle	Outer	Deep	Total	Inner	Middle	Outer	Deep
1	Deer Creek	24.1	ns	ns	ns	ns	ns	ns	ns	ns	ns
2	Deep Hole	113.4	8	12	12	**	32	0.22	0.33	0.33	0.11**
3	Leo Carrillo	95.4	12	13*	8		33	0.38	0.38	0.25	
4	Nicholas Canyon	49.2		12	12		24	0.00	0.50	0.50	
5	Encinal Canyon	96.2	8	16			24	0.33	0.67	0.00	
6	Point Dume	1.7	8	8	8		24	0.33	0.33	0.33	
7	Little Dume	214.9	12	12	12	**	36	0.25	0.25	0.25	0.25**
8	Escondido	75.6	12	12			24	0.50	0.50		
9	Malibu Point	61.2	ns	ns	ns	ns	ns	ns	ns	ns	ns
10	Topanga	223.4	ns	ns	ns	ns	ns	ns	ns	ns	ns
11	Big Rock	2.6	4				4	1.00	0.00	0.00	
12	Flat Rock	80.8	12	12	8		32	0.38	0.38	0.25	0.00
13	Ridges	213.0	16	16	16	16	64	0.25	0.25	0.25	0.25
14	Rocky Point	240.0	16	16	16	16	64	0.25	0.25	0.25	0.25
15	Resort Point	116.1	8	12	12	8	40	0.20	0.30	0.30	0.20
16	Point Vicente	35.8	16	16	16	8	56	0.29	0.29	0.29	0.14
17	Long Point	30.0	12	12	8	**	32	0.33	0.33	0.22	0.11**
18	Abalone Cove	87.6	ns	ns	ns	ns	ns	ns	ns	ns	ns
19	Bunker Point	55.7	4	4	4		12	0.33	0.33	0.33	0.00
20	Three Palms	161.6	20	20	20	12	72	0.28	0.28	0.28	0.17
21	Whites Point	124.4	8	8	8		24	0.33	0.33	0.33	
22	Point Fermin	143.5	8	8	8		24	0.33	0.33	0.33	
	**Total**	**2246.2**	184	196	168	60	621				

Area of natural rocky reef habitat <30 m depth, summary of fish sampling in each depth zone and estimated proportion of each reef in each depth zone. ns: reefs were not sampled for fishes. Blank cells indicate that rocky reef habitat was not present in that depth zone during fish sampling.

*An additional transect was performed during a sampling event, however, this does not reflect a higher proportion of reef in that depth zone.

**Depth zone was present at some or all fish sampling sites, but not sampled. In these cases the fish metrics for the outer zone were used to calculate reef specific metrics.

Kelp Bass (*Paralabrax clathratus*) and California Sheephead (*Semicossyphus pulcher*) are economically and ecologically valuable rocky reef fishes in southern California [12], [18]–[20], making them indicator species for evaluating spatial resource management actions in this region [13], [21], [22]. There has been substantial empirical work documenting patterns in life history and recruitment for both Kelp Bass [18], [23]–[29] and California Sheephead [14], [30]–[37]. Additionally, it has been demonstrated that the numerical, biomass and egg production density of both species have increased within MPAs in southern California [12], [13], [22]. However, how much individual reefs contribute to a regional estimate of fish abundance and egg production remains largely unexamined. Improving the accuracy of habitat area estimates and their associated fish abundance and egg production could help refine oceanographic based models of larval export and connectivity which may be associated with fisheries benefits from marine protected areas, e.g., [38], [39]. Further, incorporating more spatially explicit information into management strategies can increase the economic value of a fishery [40]. Therefore, refining our ability to map important rocky reef habitats and estimate their contribution to regional abundance and egg production is of high importance.

The objectives of this study were to integrate multiple spatial datasets, aerial and satellite photography, underwater field observations and expert judgment into a GIS database to produce fine scale maps of the extent of nearshore subtidal rocky reef habitat in the Santa Monica Bay region. We then applied these maps, by combining them with available fish density and size structure data from a concurrent comprehensive monitoring program, to explore the impact of habitat area on the relative contribution of individual reefs to a regional estimate of standing stock and reproductive output of California Sheephead and Kelp Bass. Understanding the potential of each area will help target and better generate expectations for fisheries management and habitat restoration efforts. Finally, we estimated the area of this important nearshore rocky reef habitat in each of the recently designated MPAs in the Santa Monica Bay region and the associated annual egg production.

## Methods

### Mapping

The study area was bounded by the coastline extending from Point Fermin northwest to Deer Creek (Figure 1), and seaward to the 30 m isobaths. The geographic extent of marine rocky reef was mapped by combining several different spatial datasets into a preliminary habitat data layer. This layer was then validated and refined using underwater field observations and expert judgment. Initial mapping and spatial analysis were done using ArcGIS software. Spatial data layers were created and maintained in the shapefile format, using the UTM Zone 11 North, WGS84 projection to minimize distortion in both area and length measurements. For geographic context and to provide a landward boundary to the mapping and spatial analysis, a shoreline spatial data layer was constructed by combining the two shoreline data layers from the US Geological Survey Coastal and Marine Geology Program Internet Map Server [41]. The shoreline layer for 1998 comprises most of the shoreline for the study area, but contains gaps, which were filled using data from the 1971-76 shoreline data layer. The 30 m isobath was mapped by extracting this feature from the bathymetric contour spatial dataset of Kelner et al. [42].

**Figure 2:**
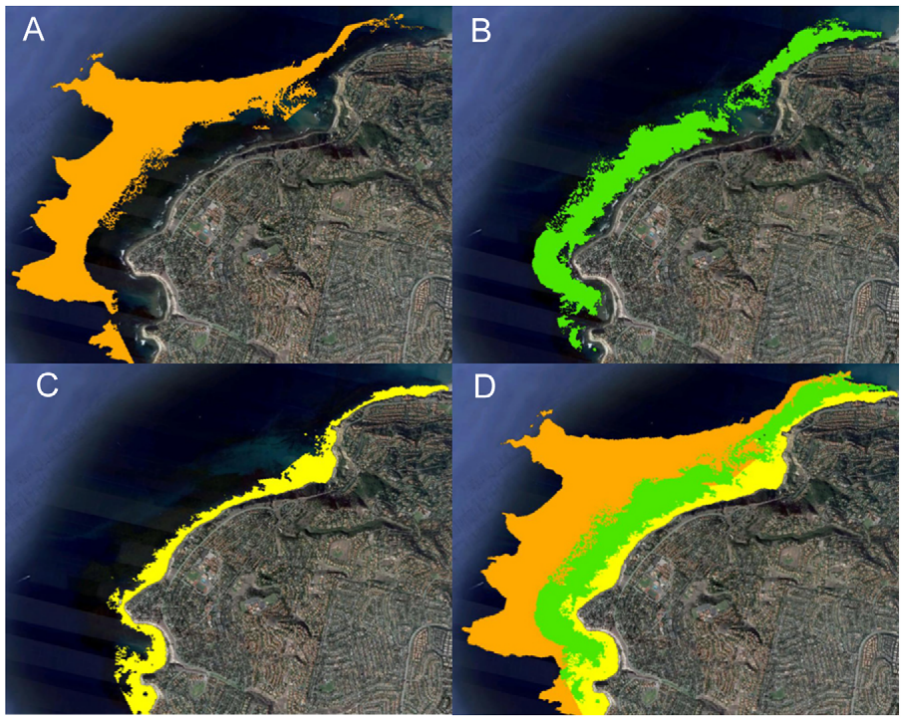
Habitat layers at Ridges and Rocky Point reefs. Habitat layers at Ridges and Rocky Point reefs using kml file displayed with Google Earth™. (A) Orange – side scan sonar - Habitat Classification. (B) Green – Kelp Canopy. (C) Yellow – course scale habitat and previously unmapped shallow rocky reef habitat. (D) All three layers combined exhibiting total extent of rocky reef habitat which is displayed for the region in Figure 1.

Initial mapping of reef extent to create the preliminary habitat map was accomplished by combining three existing vector polygon spatial data sets. The first data set was Habitat Classification (shapefile, polygon) derived from side scan sonar surveys from the Sea Floor Mapping Lab at California State University Monterey Bay (http://seafloor.csumb.edu/SFMLwebDATA.htm). There was no coverage for this data in the portion of the study area from Point Dume northwest to the study area boundary, or in the central portion of the study area from Topanga to Flat Rock (Figure 1). From this data layer, only those habitat types that correspond to or function as reef were selected. These habitat types included: deformed hummocky bedrock, differentially eroded deformed bedrock, hard anthropogenic mounds, hummocky bedrock, hummocky sediment covered deformed bedrock, mixed bimodal sediment over bedrock, mixed sediment and flat bedrock, scoured boulders and pinnacles and volcanic rock. An example of this layer can be seen in Figure 2A. The second data set was Kelp Canopy (shapefile, polygon), a highly precise polygon spatial layer created by using a 2-meter rectangular grid to classify georeferenced aerial photography [42]. As this data layer depicts the kelp canopy (primarily *Macrocystis pyrifera*), the use of the data to map marine hard bottom capable of supporting kelp holdfasts introduces some error into the analysis. Three years (1989, 1999 and 2002) of data were used in an effort to account for some of the annual variation in kelp canopy. An example of this layer can be seen in Figure 2B. The final data set used for the preliminary habitat layer was the coarse-scale mapping of hard bottom (shapefile, polygon) between the 10 and 30 m isobaths from Kelner et al. [42]. This layer only discriminates between soft sediment and hard bottom, and lacks spatial resolution in identifying boundaries between these two bottom types. It was used primarily to verify bottom type in areas not covered by the above two datasets. The layers were merged using a GIS union to create a single spatial data layer, the preliminary habitat map, retaining reference to the source data in the attribute table of the resulting data layers derived from this data layer.

As the preliminary habitat map was constructed of spatial data that does not cover the entire study area, mapping was completed and validated by a variety of techniques. To verify the accuracy and coverage of the spatial data layer, two methods were used. The first was to transform the combined spatial data layer into the Google Earth™ .kml format, and examine the mapped habitat against the georeferenced aerial imagery that serves as a back coverage in the Google Earth™ application. The photo coverage used to augment and correct the GIS union spatial data layer is the TeleAtlas March 2007 imagery available for use with Google Earth™. This technique was also used to map bottom habitat directly for the shallow zone in areas and depths where the side scan sonar survey did not collect data, and the water was shallow and clear enough to see habitat variation in the aerial photographs. The aerial imagery showed numerous additional nearshore areas were composed of hard bottom, and that the kelp canopy was more extensive than depicted in the kelp canopy data. These additional marine hard bottom areas were mapped by hand-digitizing polygons from the Google Earth™ imagery registered to the GIS spatial data. These data corrections were added to the 3rd preliminary habitat spatial data layer (Figure 2C), again retaining data source information using the polygon attributes.

In addition to verification using aerial imagery, the observed bottom characteristics during fish sampling (described below) were compared with the corresponding point locations on the preliminary habitat map. All maps were then reviewed and checked for accuracy by scientific divers who have extensive experience in the area. Finally, specific reefs were delineated and named by best professional judgment as known areas of coastline of similar contiguous habitats (Figure 1). In ArcGIS, we then calculated the total area of natural rocky reef habitat <30 m depth for the 22 reefs in the region (Table 1). The map figures shown here were produced with the PBSmapping package in R [43] using the ArcGIS created Shapefiles.

### Fish abundance and egg production potential

California Sheephead and Kelp Bass density and size structure were extracted from data collected from 2007 through 2009 following a standardized comprehensive community monitoring survey protocol; for more details on the protocol see [13]. Fish transects consist of 30×2×2 m replicate portions at multiple levels in the water column: bottom, midwater, and kelp canopy (when present). To convert to densities per m^2^ of sea floor, abundance per transect was summed across all levels and divided by 60. Divers counted and estimated total length (TL) of small fish (<15 cm TL) to the nearest cm, and larger fish (>15 cm) to the nearest 5 cm interval. Some California Sheephead tend to follow divers, while Kelp Bass appear to be repelled by divers in some circumstances and attracted to divers in others. This was accounted for by using only highly trained divers that count each fish once, always looking forward on the transect, and not counting fish that come up from behind [1].

At each site, transects are laid out in a stratified random design, with 4 transects located in each of four depth zones: inner (target depth 5 m; actual surveyed depths 3 to 8 m), middle (target depth 10 m; actual surveyed depths 7 to 13 m), outer (target depth 15 m; actual surveyed depths 11 to 19 m), and deep (target depth 25 m; actual surveyed depths 18 to 30 m). Only depth zones containing rocky reef habitat at each site are sampled, thus for a site containing all four depth zones, 16 fish transects (each with a benthic, midwater and canopy portion) would be conducted on a sampling occasion. Canopy portions were only surveyed when giant kelp canopy was present and otherwise it was observed that Kelp Bass and California Sheephead were not present along the ocean surface in the absence of kelp canopy during these surveys. Additionally, for shallow (<5 m depth) inner zone transects, where midwater and canopy portions overlapped considerably, midwater portions were not surveyed (n = 48). Three meters of visibility was the minimum threshold for sampling.

**Figure 3:**
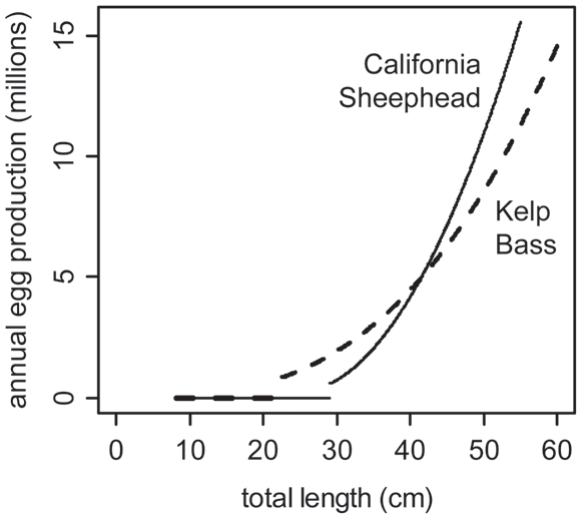
Relationship between length and annual egg production for California Sheephead and Kelp Bass. Relationship between total length and annual egg production for individual California Sheephead (*Semicossyphus pulcher*) (solid line) and Kelp Bass (*Paralabrax clathratus*) (dashed line) calculated using sources in Table 2.

Annual egg production was estimated for each Kelp Bass and California Sheephead observed using a method following Tetreault and Ambrose [12]: the product of length specific batch fecundity and mean spawning events per year for all mature (individuals ≥ length at 50% maturity) fish. However, to calculate the length specific batch fecundity relationship (Figure 3), we used updated life history parameters where available [18], [29], [36], [37], [44], [45]; see Table 2 for source details for each parameter. Also, an error was discovered and corrected in the size-specific batch fecundity relationship for Kelp Bass from Oda et al. [45]. Also, since Kelp Bass sex was not identified during surveys (while it was for California Sheephead), annual fecundity was estimated for all Kelp Bass and then multiplied by an estimate of female:male sex ratio (Table 2).

**Table 2:** Life history relationships used to calculate size-specific annual fecundity relationships.

Species	Relationship	Source
Kelp Bass	*Total length (TL) to standard length (SL) conversion:*	[18]
*(Paralabrax clathratus)*	SL [in mm] = 0.83*TL [in mm] - 1	
	*Batch fecundity function (hydrated oocytes; HO):*	1 [45]
	HO = 0.00381*SL^2.93^[in mm]	
	*Mean number of spawning events per year:* 47	[44], [45]
	*Length of size class with 50% mature:* 226 mm TL	[18]
	*Sex ratio (female:male):* 0.45	[29]
California Sheephead	*Total length (TL) to standard length (SL) conversion:*	[36]
*(Semicossyphus pulcher)*	SL [in mm] = 0.80*TL [in mm] + 3	
	*Batch fecundity function (hydrated oocytes; HO):*	[37]
	HO = 3.0041*SL^2^[in mm] - 1199.6*SL[in mm] + 122526	
	*Mean number of spawning events per year*: 86	[44]
	*Length of size class with 50% mature (for fish collected from Palos Verdes)*: 291 mm TL	[36]

1 The relationship reported in Oda et al. (1993) was using the Log (10) scale, however, the actual regression (as evident in their Figure 6) was fitted with natural log transformations. The corrected relationship we used is reported here.

Total numerical abundance and annual egg production (with associated 95% confidence intervals) for each reef and for the region was calculated, based on a depth zone stratified sampling approach, following McCormick and Choat [46]. At each sampling location, only depth zones where rocky reef habitat was present were sampled. Therefore, we estimated the proportion of area of each reef in each depth zone by dividing the number of fish transects in each depth zone by the total number of transects for that reef. Young-of-the-year fish (individuals <10 cm TL), were removed prior to analysis to decrease bias in abundance estimates associated with timing of surveys relative to local recruitment events.

The influence of depth zone on abundance of each species per transect was investigated with a model selection approach using AIC [47], [48]. For each species, two models were compared: model 1 estimated a depth zone specific mean for abundance per transect and model 2 estimated a single mean abundance per transect for data from all depth zones pooled. Both assume a negative binomial probability distribution with a log-link function. Models were fitted and 95% likelihood profile confidence intervals were calculated using the glm.nb function in R [43], [49]. Parameter estimates and confidence intervals were back transformed and converted to densities prior to plotting.

### Recently designated MPAs in the region

Using ArcGIS, we calculated the total area of natural rocky reef habitat <30 m depth in each of the four MPAs in the region that were adopted by the California Fish and Game Commission on 15 December 2010, to be implemented 1 January 2012 (MPA boundaries can be found at: http://www.dfg.ca.gov/mlpa/pdfs/sc_boundaries.pdf). We also estimated the contribution of this habitat, in each of the MPAs, to regional abundance and annual egg production. This was done by multiplying the percentage of reef area in each MPA by the total abundance or annual egg production for each reef and dividing by an estimate of total abundance or annual egg production for the region. For this calculation we estimated the regional totals including the un-surveyed reefs by assuming mean density or egg production for all un-surveyed reefs.

## Results

### Mapping

The extent of the nearshore subtidal rocky reefs was mapped (Figure 1).The three data layers used to generate the map (for an example see Figure 2) are available for download as ESRI© format shapefiles and Google Earth™ compatible kml files from the Vantuna Research Group website (http://college.oxy.edu/vrg). There are approximately 2246.2 hectares of nearshore (<30 m depth) natural rocky reef habitat in the study region (Table 1). The largest reef is Rocky Point comprising 240.0 hectares. The smallest reefs are Big Rock and Pt. Dume, comprising 2.6 hectares and 1.7 hectares, respectively. Big Rock is a single patch reef in the middle of the large Topanga reef complex (Figure 1B). Big Rock was delineated as a separate reef since it was the only part of Topanga surveyed for fishes, and therefore sufficient data was not available to estimate fish metrics for the entire Topanga reef complex. Rocky reef was found at all fish sampling sites, corroborating the mapping data. However, based on observations from experts, a small number of minor anomalies were noted in the preliminary habitat map in offshore areas that represented missing data. These were subsequently corrected and included in the 3rd preliminary habitat spatial data layer (Figure 2C).

### Fish abundance and egg production potential

A total of 621 fish transects were completed across 18 of the 22 reefs in the region (Table 1). Between one and five sites were sampled at each reef, with between four and 16 transects surveyed per site, depending on the number of depth zones present. In most cases, sampling effort was proportional to reef area, however, sampling conditions influenced the final distribution. All 621 transects included a bottom portion, while 571 included a midwater portion and 395 transects included a canopy portion. Ninety-nine percent of California Sheephead were observed on the bottom portions of transects. For Kelp Bass, 77% were observed on the bottom, 20% on the midwater and 3% on the canopy portions of transects.

**Figure 4:**
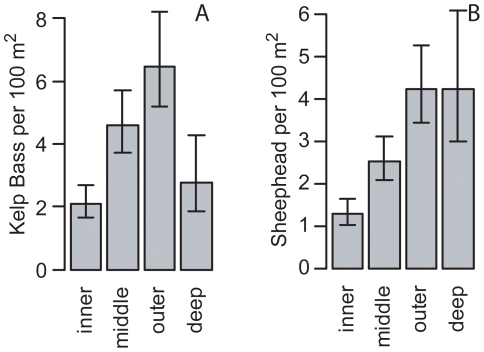
Depth zone specific density patterns of Kelp Bass and California Sheephead. Depth zone specific density of (A) Kelp Bass (*Paralabrax clathratus*) and (B) California Sheephead (*Semicossyphus pulcher*) for rocky reefs in Santa Monica Bay, California. Error bars are 95% likelihood profile confidence intervals.

Depth zone exhibited a clear effect on abundance per transect of California Sheephead and Kelp Bass based on AIC differences and 95% confidence intervals (Figure 4). Models that estimated depth zone specific means had substantially lower AIC values (51 and 40 for California Sheephead and Kelp Bass, respectively) than the models that did not include the effect of depth zones. An AIC difference greater than 2.0 can be interpreted as equivalent to a statistically significant result [47]. California Sheephead were most abundant along outer and deep depth zone transects, and lowest in the inner depth zone. Kelp Bass were two to three times more abundant in the outer zone than in the inner and deep zones, while middle zone transects had a moderate abundance per transect.

The reefs that were surveyed for fishes had a total area of 1850 ha (18,498,190 m^2^) comprising 82% the natural rocky reef habitat <30 m depth that was mapped in the region (Table 1). For this area of reef, California Sheephead (>10 cm TL) had a total abundance of 451 thousand fish (95% CI: 369 to 533 thousand) and an annual egg production of 203 billion eggs (95% CI: 135 to 272 billion). Kelp Bass (>10 cm TL) had a total abundance of 805 thousand fish (95% CI: 669 to 941 thousand) and an annual egg production of 512 billion eggs (95% CI: 414 to 610 billion). Subtle differences between the species-specific relationship between body length and annual egg production (Figure 3) resulted in a substantial difference between their annual egg production estimates for the region. While there are 80% more Kelp Bass on these reefs, they produce 152% more eggs annually. Even though large California Sheephead females (>45 cm TL) produce more eggs per unit length than large Kelp Bass females, most fish in the region are in the smaller 20–40 cm TL size range, where Kelp Bass are estimated to produce more eggs annually per unit length.

**Figure 5:**
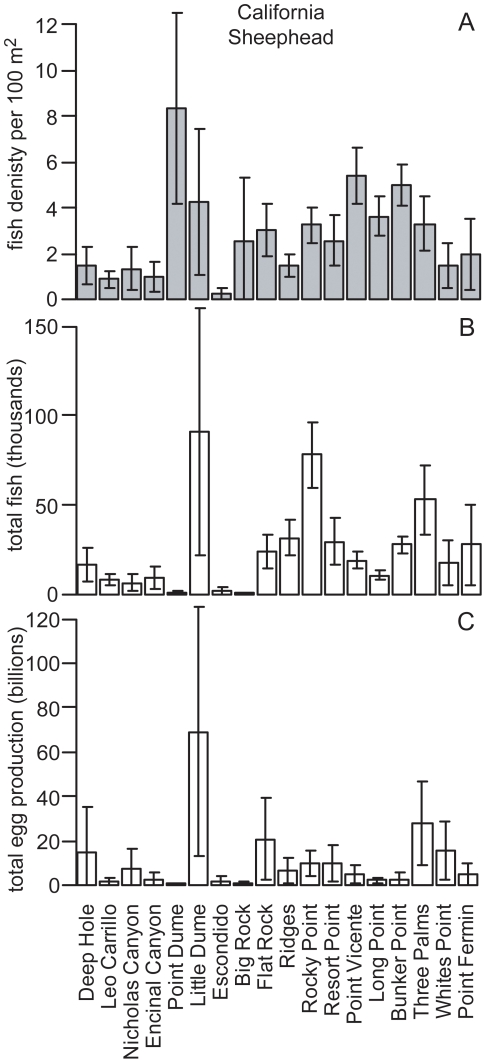
California Sheephead density, abundance and egg production. California Sheephead (*Semicossyphus pulcher)* (A) mean numerical density, (B) total abundance and (C) total egg production for each reef that was surveyed in Santa Monica Bay, California. Error bars are 95% confidence intervals.

**Figure 6:**
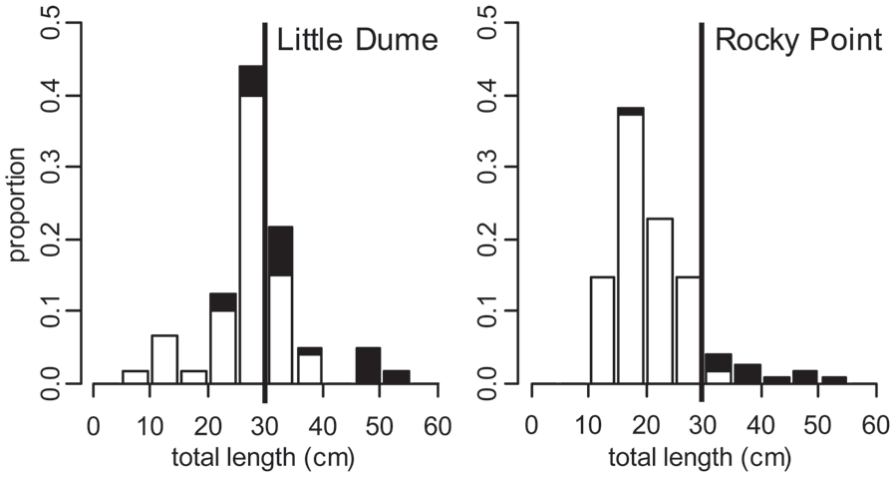
California Sheephead size structure at Little Dume and Rocky Point. California Sheephead (*Semicossyphus pulcher*) size structure (proportion of individuals per 5 cm size class) of females (white) and males (black) for Little Dume reef and Rocky Point reef. The legal minimum size limit (30 cm) for recreational fisheries is indicated by a black line.

**Figure 7:**
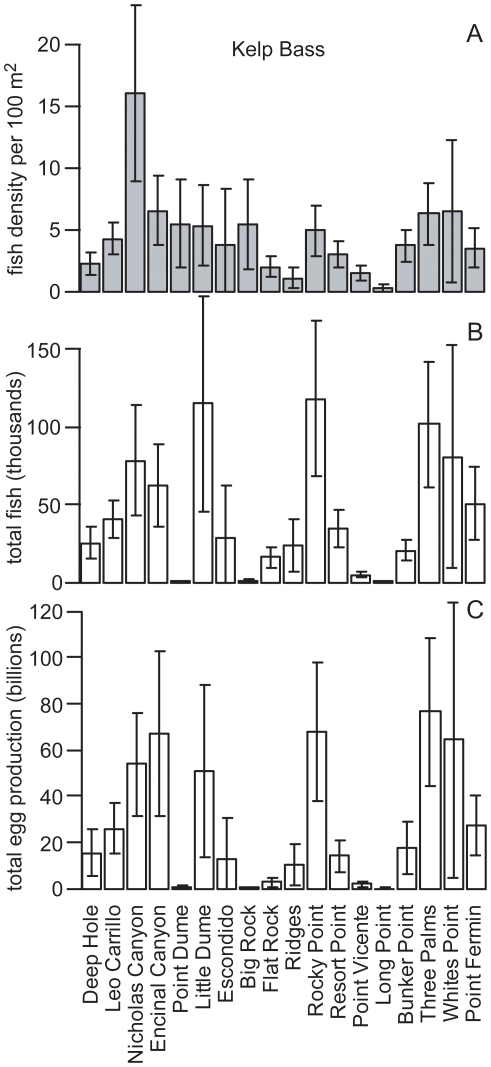
Kelp Bass density, abundance and egg production. Kelp Bass (*Paralabrax clathratus*) (A) mean numerical density, (B) total abundance and (C) total egg production for each reef that was surveyed in Santa Monica Bay, California. Error bars are 95% confidence intervals.

**Figure 8:**
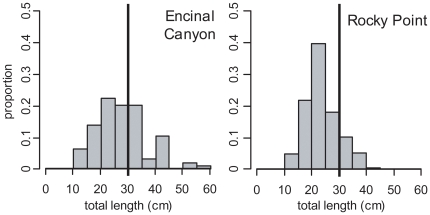
Kelp Bass size structure at Encinal Canyon and Rocky Point. Kelp Bass (*Paralabrax clathratus*) size structure (proportion of individuals per 5 cm size class) for Encinal Canyon reef and Rocky Point reef. The legal minimum size limit (30 cm) for recreational fisheries is indicated by a black line.

Both species presented clear examples of how reef area and fish size structure can influence a single reef's contribution to regional egg production. For California Sheephead, Point Dume had the highest density (Figure 5). However, given its small reef area (1.7 ha), its contribution to regional abundance and egg production was negligible. Little Dume and Rocky Point, with relatively high densities, and being two of the largest reefs in the region (214.9 ha and 240.0 ha, respectively), had the highest California Sheephead abundance (90 thousand and 77 thousand fish, respectively). However, due to differences in size structure, with Rocky Point having a greatly reduced proportion of larger females beyond the legal recreational size catch limit (Figure 6), its contribution to regional egg production was relatively minor. While Little Dume, with a relatively larger proportion of females in the 30 to 40 cm TL range, just above the legal size limit, had seven times the annual egg production of Rocky Point, producing 34% of the regional egg production even though it represents only 12% of the reef area surveyed. For Kelp Bass, six medium to large reefs made substantial contributions to regional egg production (Figure 7), from which there were further examples that demonstrated the influence that size structure could have on egg production. Encinal Canyon has only 40% of the reef area (Table 1) and about 50% of the Kelp Bass abundance (Figure 7) of Rocky Point. Yet, since Encinal Canyon had a higher proportion of larger fish above the recreational legal size limit (Figure 8), the two reefs had almost identical estimates of mean annual egg production (67 and 68 billion eggs, respectively).

### Recently designated MPAs in the region

**Figure 9:**
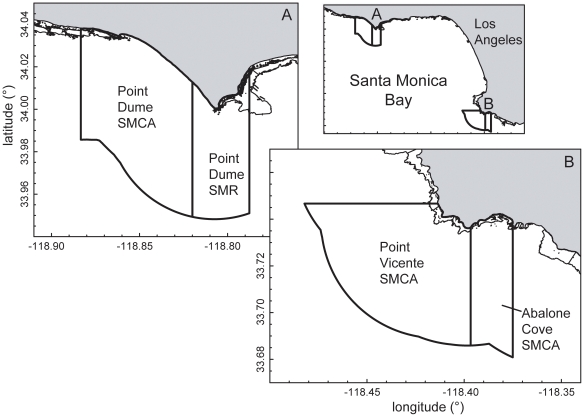
Map of nearshore rocky reef habitat in Santa Monica Bay MPAs. Map of the extent of nearshore rocky reef habitat in each of the MPAs designated in (a) Malibu and (b) the Palos Verdes Peninsula.

Small percentages (1 to 5%) of the total area of each of the four MPAs designated in Santa Monica Bay contain the rocky reef habitat <30 m depth mapped in this study (Table 3, Figure 9). Once the MPAs are implemented, at least some types of fishing will be excluded in 10.9% of the rocky reef habitat mapped in the study region. Each MPA will represent a relatively small percentage of the regional egg production (based on the 2007-2009 data available for this study), with the exception of California Sheephead in Point Dume SMR. The mapped habitat in Point Dume SMR is mostly the Little Dume reef which had the highest annual egg production for the region.

**Table 3:** Habitat area and egg production in Santa Monica Bay MPAs.

	Rocky Reef Area (ha)	Percent of MPA Area	Percent of Regional Mapped Habitat	Percent of Regional Sheephead Egg Production	Percent of Regional Kelp Bass Egg Production
Point Dume SMCA	39.3	1.0%	1.7%	0.4%	4.4%
Point Dume SMR	95.7	4.9%	4.3%	12.5%	3.7%
Point Vicente SMCA	72	1.8%	3.2%	3.2%	0.6%
Abalone Cove SMCA	37.9	3.1%	1.7%	1.2%	0.04%
**Total**	244.9	10.8%	10.9%	17.2%	8.7%

Amount of mapped habitat (i.e. nearshore < 30 m depth natural rocky reef) and estimated annual egg production in recently designated MPAs in Santa Monica Bay.

## Discussion

This study produced the first comprehensive map of nearshore rocky reef habitat and associated area estimates of habitat <30 m depth for the Santa Monica Bay region. Applying these measurements of reef area, with available fish density and size structure data for two important recreational fish species, provided a regional estimate of abundance and annual egg production against which contributions of individual reefs could be measured. Results demonstrated California Sheephead residing in relatively small fractions of the regional reef area (ca. 10%) can potentially produce a substantial proportion of the regional egg production (>30%). While this critical habitat makes up only a few percent of the newly designated MPAs in the region, results provide some optimism that regional fisheries could benefit through an increase in overall reproductive output, if adequate increases are realized in the size structure of targeted species.

The mapping process proved effective in identifying and delimiting rocky reef habitat in areas, and at depths, where no side-scan data were available. Fine scale maps of rather complex habitats were produced for both areas where there was substantial shallow rocky reef habitat at the shoreline (e.g., along the Palos Verdes Peninsula) and areas where there was mid-depth rocky reef along a sandy shoreline (e.g., along Malibu). The spatial extent of giant kelp canopy served as a useful proxy for rocky reef across most of the depth range covered in the study, while satellite imagery proved valuable for mapping shallow habitat. As high quality satellite images become freely available through sources like Google Earth™, this method is particular applicable and cost effective for delimitating the extent of general habitat types (e.g., hard vs. soft bottom) along coastlines where other spatial habitat data is unavailable. Additionally, recent research suggests that measurement of giant kelp canopy area from satellite imagery can also serve as a good proxy for the underlying biomass of giant kelp [50], which, along with other metrics of habitat quality, could be incorporated into future evaluations of rocky reef productivity.

Estimates of abundance and egg production produced for this study are likely conservative for both species. First, these estimates did not include the 18% of rocky reef habitat that was mapped but not been surveyed for fishes. The same applies for the artificial reef structures (e.g., quarry rock breakwaters) located predominantly along the sandy bottom portion in the center of Santa Monica Bay. These include nine artificial reefs of various designs created from 1960 to 1987 which occupy 114 hectares [51], representing an additional 5% of rocky reef habitat in the study region. Artificial reefs in this region tend to function similarly to natural reefs [52]–[54]. Therefore, these discrepancies could be accounted for by increasing the estimates by 23%, equivalent to applying the mean values for the various metrics across the un-surveyed natural and artificial reefs in the region. For other factors which could contribute to an underestimate of total abundance and egg production, there currently does not appear to be straightforward methods to account for them in our estimates. The first of these factors is that both species, but particularly California Sheephead, are known to reside at depths below 30 m. Kelp bass are primarily limited to depths of 3 to 25 m [55] with maximal densities found in this study at 7 to 19 m depth. California Sheephead have been observed down to 85 m [56] and were most abundant at the deeper depths of this study. However, for this region, most of the natural nearshore rocky reef habitat becomes soft bottom prior to reaching 30 m depth (Figure 1), which should minimize the impact of this factor. Second, except for inner zone transects, the entire water column was not surveyed. This probably has a negligible impact on California Sheephead estimates, since 99% of California Sheephead were observed along the bottom. However, 20% of Kelp Bass were observed on the midwater transect portions, and, at deeper depths, a substantial fraction of the midwater was not sampled nor included in the density estimates.

The precision of reef-specific fish metrics, particularly egg production potential, was relatively low for some reefs as evident by the large 95% confidence intervals. However, the method we followed to make these estimates [46], based on a depth-stratified random sampling approach, was supported by the clear influence that depth zone had on both California Sheephead and Kelp Bass density. This approach may also be useful with other fishes as depth specific habitat use appears to be common among temperate reef fishes [1], [57]. The high level of variance was due to the high spatial heterogeneity and relatively low abundance, to some level attributable to micro-habitat preferences of both species within rocky reefs [28], [34], [58]. Variation was further amplified in the estimation of annual egg production due to the exponential relationship of body size and fecundity which, for some reefs, led to a large confidence interval.

Future efforts to quantify total abundance could benefit from stratifying a greater number of random samples amongst rocky reef micro-habitats and optimally allocating effort to those according to habitat specific variance. This would likely increase accuracy, precision and sampling efficiency [46], [59]. The physical structure and biological habitat components (e.g., giant kelp density) of reefs are likely to explain aspects of spatial variability in the distribution and abundance of fishes and invertebrates on temperate rocky reefs [1], [57], [58], [60]–[63]. The fish sampling protocols which generated the data for the present study were designed for monitoring changes over time [13], and their use is likely to expand with the new network of protected areas being implemented in the region. Given the spatial extent of rocky reefs in southern California, let alone Santa Monica Bay, implementing modifications of existing methods would come with a considerable financial burden and likely result in trade-offs between efficiency and performance depending on the goals of the monitoring program and biological differences in species of interest [59].

While the data available may have limited the precision at which estimates of some reef-specific metrics were made, combining reef area estimates with fish density, size-structure and size-fecundity relationships still provides useful insight into the potential value of specific reefs from a regional-scale perspective in terms of standing stock and reproductive output. While the smallest reefs in the region (Big Rock and Point Dume) had some of the highest density values for both species, from a regional fisheries perspective, they have limited value. On the contrary, some of the largest contiguous reefs may have lower densities, but as a whole, can contribute substantially to regional reproductive output. Therefore, it is important that the extent of appropriate habitat is considered when resource management actions are being planned and evaluated.

The size structures of both focal species in this study illustrate impacts from fishing. Throughout southern California, commercial and recreational fishing pressure on California Sheephead has reduced the prevalence of larger females, as well as impacted other life history characteristics [14]. Size structures for both species from reefs in this study appear very similar to those from other areas open to fishing around southern California [12], [27], [36]. Reefs easily accessible to recreational anglers originating from King Harbor, that are known to experience high levels of fishing pressure (e.g., Rocky Point), exhibited even more pronounced effects in terms of the proportion of individuals above the minimum legal size catch limit (Figure 6, 8).

Size structure was a key factor in how much reefs contributed to the regional egg production. Further, results demonstrate the potential that relatively small increases in the proportion of females in the larger size classes on larger reefs could have on regional egg production, due to the exponential relationship between body length and annual fecundity (Figure 3). For California Sheephead, a single large reef (Little Dume) which represents only 12% of the total habitat of reefs that were surveyed, accounted for 34% of the regional annual egg production. Therefore, there is potential that the new MPAs in the study region, while only prohibiting fishing in 10.9% of the rocky reef habitat mapped for this study, could have a positive fisheries impact by increasing the regional egg production. Over the next decade it is reasonable to conclude that the prevalence of large females in MPAs will increase substantially based on what has been observed in existing MPAs in California [12], [13], [21], [22], [64] and around the world [65], [66].

Increases in density, biomass and age structure of fish populations over time within the new MPAs may also result in changes to other life history characteristics that have the potential to negatively or positively impact their overall reproductive success. Observations of fish populations across spatial or temporal gradients of density and/or biomass suggest that, as density and/or biomass builds within MPAs, fishes are likely to experience some level of density dependence in somatic growth, with evidence to date being most clear for juvenile stages [67]–[69]. While this would tend to reduce the per-capita increase in annual egg production, density related effects are likely to be mitigated, to some degree, by spillover (i.e. movements of fishes out of MPAs), for which there now appears to be substantial evidence [70]. Further, high fishing pressure over the past few decades decreased the sizes and ages at maturity and sex change in populations of California Sheephead in southern California [14], diminishing female reproductive output [36]. Therefore, one could expect the reverse to occur when the effects of fishing are removed in MPAs. Finally, it has been demonstrated in other temperate fishes, that individuals that are older, larger and/or have more spawning experience can produce more viable eggs and larvae or have higher fertilization rates, resulting in greater reproductive success [71], [72]. It is not clear if, or to what degree, changes in these in these life history characteristics will occur in populations of Kelp Bass and California Sheephead associated with these MPAs. This will require further empirical research, and the MPA network in California may provide such an opportunity.

Given the multiple anthropogenic stressors southern California's urban reefs experience and declines in recreational and commercial fisheries [6]–[10], [14], [17], [73], a broad suite of management strategies will be needed to successfully manage marine resources in the region. These should include marine protected areas and other top-down species-specific strategies for managing fishing pressure, such as adjusting total allowable catch and increasing legal size limits [36], efforts likely to help reduce impacts on size structure [14]. Management should also include localized efforts to improve abiotic and biotic aspects of habitat quality. Kelp restoration through urchin relocation has proved effective, resulting in rapid and resilient increases kelp density on previously barren reefs [74]. Data products from the present study will also be informative for future artificial reefs projects that have the goal of fishery production. Mapping efforts such as this provide valuable information (e.g., extent of critical habitats) that can be incorporated into planning, setting exceptions for and evaluating resource management actions. Moving forward, further research is needed to better account for micro-habitat differences in abiotic and biotic habitat quality (e.g., reef relief, substrate, and macroalgal density). This may improve precision when estimating fish abundance and reproductive output metrics, and better focus spatial resource management efforts on areas with the greatest need and potential to produce positive outcomes.
